# Spatiotemporal Dynamics of Microbial and Fish Communities in the Thracian Sea Revealed by eDNA Metabarcoding

**DOI:** 10.3390/microorganisms13102373

**Published:** 2025-10-15

**Authors:** Maria Tokamani, Panagiotis Liakopoulos, Konstantinos Tegopoulos, Aristea-Marina Zigkou, George Triantaphyllidis, Nikolaos Kamidis, Maria E. Grigoriou, Raphael Sandaltzopoulos, Petros Kolovos

**Affiliations:** 1 Department of Molecular Biology and Genetics, Faculty of Health Sciences, Democritus University of Thrace, 68100 Alexandroupolis, Greece; tokamanimaria@hotmail.com (M.T.); pliakopo@mbg.duth.gr (P.L.); ktegopou@mbg.duth.gr (K.T.); marina_zigkou@outlook.com (A.-M.Z.); mgrigor@mbg.duth.gr (M.E.G.); 2 MINDS Technologies & Environmental Sciences PC, 19500 Lavrio, Greece; georgetrianta@hotmail.com; 3 Hellenic Agricultural Organisation ELGO-DIMITRA, Fisheries Research Institute, 64007 Kavala, Greece; nikkami@elgo.gr

**Keywords:** eDNA, metabarcoding, microbiome, fish communities, metagenomic

## Abstract

The Thracian Sea, a semi-enclosed coastal basin in the northeastern Aegean Sea, represents a dynamic marine environment influenced by freshwater inputs, stratification, and seasonal variability. Here, we investigated the spatiotemporal dynamics of microbial and ichthyofaunal communities using environmental DNA (eDNA) and high-throughput sequencing across various stations in the vicinity of the Thracian Sea, in consecutive months (through spring and summer). Seawater samples were collected from the surface and thermocline layers, and environmental parameters were recorded to examine their influence on biodiversity patterns. Microbial communities exhibited strong seasonal and depth-related structuring. Alpha diversity was highest in spring and declined during summer, while beta diversity analyses revealed clear clustering by month and depth. Dominant taxa included Alphaproteobacteria (SAR11), Cyanobacteria (*Synechococcus*, *Prochlorococcus*), with distinct core microbiomes. Fish communities, identified via *CytB* metabarcoding, displayed marked temporal turnover but limited spatial segregation. While alpha diversity metrics did not differ significantly, beta diversity analyses showed seasonal shifts with dominant taxa such as *Raja* spp., *Engraulis* spp., and *Diplodus sargus*. Multivariate and co-structure analyses (Mantel, Procrustes) revealed moderate but significant concordance between microbial and fish communities and support the existence of similar biodiversity responses to environmental parameters across temporal and spatial variability. Co-occurrence networks further present depth-specific associations, with surface communities being more cooperative and phototrophic, while thermocline networks showed modularity and potential ecological specialization. This study highlights the value of integrated eDNA-based monitoring in revealing seasonal biodiversity dynamics and ecological interactions in coastal marine ecosystems, supporting future spatial planning and conservation strategies in the Thracian Sea.

## 1. Introduction

The Thracian Sea, located in the northern Aegean and bordering the region of Thrace in northeastern Greece, is a semi-enclosed marine basin, shaped by the interplay of riverine, coastal, and open sea processes [1]. It encompasses both deep offshore zones and extensive shallow coastal shelves, which are strongly influenced by freshwater inflows, particularly from the Evros and Nestos Rivers [2,3,4]. It is characterized, also, by a complex geophysical flow field due to the buoyancy outflow of the low salinity waters from the Black Sea [1,5,6,7]. In more detail, the presence of widespread plateaus in northern Samothraki, with depths of less than 100 m, in combination with the mixing of Black Sea and Aegean waters, creates a complex anticyclonic formation system, known as “Samothraki gyre”, that varies seasonally [1,7,8]. These inputs from rivers and the Black Sea transport nutrients and organic matter into the marine environment, supporting primary productivity and influencing microbial community structure [1,9], rendering the region highly sensitive to both environmental and anthropogenic influences.

The Thracian Sea (Northeast Aegean Sea) hosts ecologically significant habitats such as seagrass meadows (*Posidonia oceanica*) and brown algae (*Cystoseira* spp.), marine protected zones, and the Evros Delta [10,11]. However, these ecosystems are under increasing pressure from urban expansion, agricultural runoff, unsustainable irrigation, aquaculture and overfishing, coastal tourism, and marine traffic, further intensifying the need for continuous environmental monitoring and spatial planning [11,12], especially concerning diversity and water quality [13].

Marine microorganisms play a central role in biogeochemical cycles by regulating nutrient availability, oxygen dynamics, and ecosystem productivity. They are also highly responsive to environmental changes, providing insight into sources of organic matter and ecosystem disturbances [14,15,16,17,18,19]. In semi-enclosed basins, like the Thracian Sea, where salinity, stratification, and nutrient gradients fluctuate seasonally [1,5,6,9,18,20], microbial communities can serve as indicators of both natural variability and anthropogenic stress [13,21]. Advances in high-throughput sequencing of environmental DNA (eDNA), particularly targeting the 16S rDNA V4 region or other genetic markers, allow for high-throughput analyses of microbial and eukaryotic diversity and function [22,23,24].

On the other hand, environmental DNA (eDNA) techniques have emerged as powerful, non-invasive tools for detecting aquatic species, including elusive or migratory organisms [25,26,27]. Genetic markers such as *mtCytB* and *mt12S* (MiFish primers) are widely used to monitor fish populations and assess ichthyofaunal dynamics across marine habitats [27,28,29]. In the Thracian Sea, seasonal fluctuations in fish and cephalopod populations correlate indirectly with key physicochemical parameters like temperature, salinity, and dissolved oxygen [1,30,31].

Environmental DNA (eDNA) provides a state-of-the-art, time- and cost-efficient method, offering high sensitivity and early detection of invasive or endangered species. This powerful, non-invasive, and non-destructive approach allows for the monitoring of marine fish and microbial biodiversity, enabling the detection of multiple species from a single water sample. eDNA serves as an alternative to traditional ecological survey methods. This is especially important in regions experiencing climate and/or anthropogenic pressures or in oligotrophic and biodiverse environments such as the Thracian Sea, where traditional methods may underrepresent cryptic or low-abundance species. Approaches like metabarcoding have revolutionized biodiversity surveys by enabling high-throughput, multispecies detection [25]. This study highlights the value of integrated eDNA-based monitoring in revealing seasonal biodiversity dynamics and ecological interactions in coastal marine ecosystems, supporting future maritime spatial planning and conservation strategies in the Thracian Sea.

Despite the ecological and economic significance of the Thracian Sea, it remains understudied in terms of integrative molecular biodiversity [32]. Previous research has largely focused on either environmental variables [2,4,5,6,33] or data from marine megafauna (nekton) [30,34,35,36,37] or microorganisms [1,3,10,18,38], with few efforts combining metagenomic approaches with eDNA-based species detection [39]. Thus, this study investigated the spatiotemporal dynamics of microbial and ichthyofaunal communities using environmental DNA (eDNA) and high-throughput sequencing across various stations in the Thracian Sea over consecutive months in spring and summer. The research introduces a model for marine biodiversity monitoring to address existing gaps by examining microbial ecology and ichthyological analysis through eDNA technologies.

## 2. Materials and Methods

### 2.1. Study Area and Seawater Sample Collection

**Figure 1:**
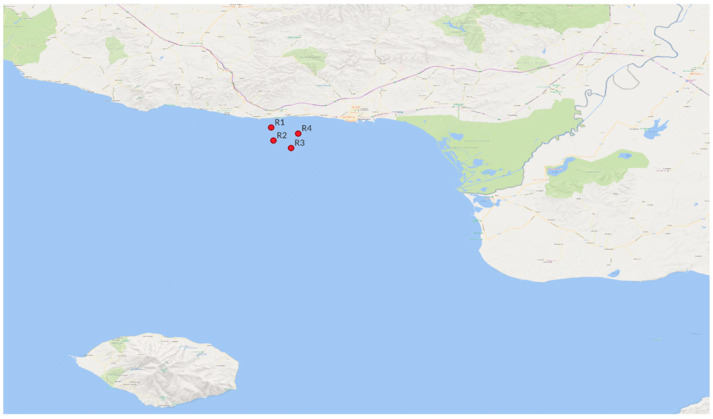
Map of Thracian Sea (Northeastern Aegean Sea), depicting the stations of the study area (R1–R4 denotes the four stations).

Seawater samples were collected from the Thracian Sea in the northern Aegean Sea as part of a pilot study from May to August 2024, aimed at developing a standardized protocol for environmental DNA (eDNA) analysis. Four sampling stations (R1–R4) were selected, with stations R1 and R4 located closer to the coast and stations R2 and R3 further offshore (Figure 1). The map presented in this work was created using QGIS (v.3.44.1) [40] with Bing Map [41] as a background layer. At each station, four liters of seawater were collected from the surface (A) and another four liters from the thermocline depth (B) using a Niskin bottle, at four time periods (May [S1], June [S2], July [S3], and August [S4] in 2024). The thermocline depth was determined in situ based on a steep temperature gradient.

Environmental parameters, including temperature, pH, conductivity, dissolved oxygen concentration, and oxygen saturation, were recorded using a multiparameter CTD detector (SBE 19plus V2 SeaCAT, equipped with an SBE 43 oxygen sensor). Metadata of the seawater samples, in more detail, are presented in Supplementary Table S1 or Supplementary Table S6. Principal Component Analysis (PCA) was applied to explore the underlying structure of environmental variables across samples and to identify major axes of variation. Quantitative environmental data were first transformed using a square root function to reduce skewness, followed by normalization (row-wise standardization to unit total) to ensure comparability across variables. PCA was performed using the *prcomp* function [42,43,44] in R with centering and scaling. To further aid interpretation, PCA biplots were constructed by overlaying environmental loadings (variable vectors) onto the ordination space. Variable arrows were scaled proportionally to reflect contribution strength and plotted alongside a correlation circle. The ggplot2 (v.3.5.2) [44], ggrepel (v.0.9.6) [45], and factoextra (v.1.0.7) [46] packages in R were used for enhanced visualization.

Filtration was employed as the primary method for capturing eDNA. A total of 1 L of seawater per sample was filtered through 45 mm EO-treated glass fiber filters (Macherey-Nagel, Düren, Germany, Cat. No. 740564) using gravity. After filtration, 5 mL of absolute ethanol HPLC Grade (100%) was passed through each filter to preserve the captured DNA, and filters were subsequently stored at −20 °C. DNA extraction was performed using the NucleoSpin^®^ eDNA Water Kit (Macherey-Nagel, Düren, Germany, Cat. No. 740402), following the manufacturer’s protocol with minor modifications adapted for marine samples. DNA concentrations were quantified using a Qubit™ dsDNA HS Assay Kit (Thermo Scientific, Waltham, MA, USA, Cat. No. Q32854).

### 2.2. Genetic Marker Amplification

Universal primers were selected based on a literature review, targeting mitochondrial gene regions commonly used for vertebrate identification, the cytochrome b (*mtCytB*, [29]) gene, as well as the V4 region [47,48] of the 16S rDNA gene for microbial profiling. Polymerase chain reactions (PCR) were conducted using 20 ng of eDNA and the high-fidelity KAPA HiFi polymerase (KAPA Biosystems, Wilmington, MA, USA, Cat No. KK2101), with optimized cycling conditions for each primer pair. All PCR reactions were performed in a 25 µL total volume mixture consisting of 1× KAPA HiFi Fidelity Buffer, 200µM dNTP mix each, 0.4 μΜ of each primer (forward and reverse), 20 ng of template DNA, and 1U of KAPA HiFi Polymerase. The thermal cycling protocol for each marker included an initial denaturation at 95 °C for 3 min followed by 35 cycles of denaturation at 98 °C for 20 s, annealing at 59 °C or 52 °C for 15 s (*CytB* and V4 pair accordingly), and extension at 72 °C for 15 s. A final extension step was performed at 72 °C for 1 min and samples were held at 4 °C until further processing. Expected amplicon length for the V4 region is 290 bp and 350–450 bp for *CytB*. PCR amplicons were assessed by 1% agarose gel electrophoresis and purified using NucleoMag^®^ magnetic beads (Macherey-Nagel, Düren, Germany, Cat. No. 744970), and amplicon concentrations were re-quantified using the Qubit system.

### 2.3. Library Construction and Sequencing

Libraries were constructed from 100 ng amplicon DNA using the NEBNext^®^ Fast DNA Library Prep Set for Ion Torrent™ (NEB, Ipswich, MA, USA, Cat. No. E6270L), following the manufacturer’s protocol. Products were barcoded using Ion Xpress Barcode Adapters™ (Thermo Scientific, Waltham, MA, USA, Cat. No. 4474517). Libraries were quantified using the Ion Library TaqMan^®^ Quantitation Kit (Thermo Scientific, Waltham, MA, USA, Cat. No. 44468802), diluted to 80 pM, and pooled for sequencing. Template preparation was performed on the Ion Torrent Chef System, followed by sequencing on the Ion Torrent S5 platform using a 530 chip (Thermo Scientific, Waltham, MA, USA, Cat. No. A34019 and A27763) at the OMIC-ENGINE Next Generation Sequencing Facility of the Democritus University of Thrace, Greece. Base-calling, barcode demultiplexing, and initial quality filtering were performed using Torrent Suite software v.5.18 (Thermo Fisher Scientific, Waltham, MA, USA) with default settings.

### 2.4. NGS Analysis

Raw BAM files from sequencing runs were converted to FASTQ format using the samtools bam2fq function [49]. Primer-specific demultiplexing was performed using the Split_on_Primer.py script (https://github.com/Y-Lammers/Split_on_Primer, accessed on 5 March 2025). The quality of the sequencing reads was assessed using FastQC (version 0.12.0, https://www.bioinformatics.babraham.ac.uk/projects/fastqc/). Downstream analysis performed using Qiime2-2024.2 [50]. Reads were trimmed using cutadapt [51] to remove low-quality bases and adapter sequences, with a minimum length threshold of 100 bp. For each marker, the expected amplicon lengths were approximately 360–480 bp for *CytB* and 280–300 bp for the 16S V4 region. Denoising and quality filtering were carried out using the DADA2 [52] pipeline implemented in QIIME 2 (version 2024.2). Primer sequences (15 bp) were trimmed from the 5′ ends of reads, and reads were truncated based on quality scores. Chimeric sequences were identified and removed using a de novo consensus approach, and the DADA2 error model was trained using one million reads with pseudo-pooling enabled. Amplicon sequence variants (ASVs) were inferred and used for downstream taxonomic and diversity analyses. A total of over 15 million raw sequencing reads were generated across the two markers (V4, *CytB*), with an average of 40,500 (V4) and 53,500 (*CytB*) reads per sample. Following quality control, read retention varied, with the V4 region retaining 46% of reads and *CytB* retaining 40%.

### 2.5. Taxonomic Assignment and Diversity Analysis

Taxonomic classification of ASVs was performed using the BLAST+ local alignment algorithm within QIIME2-2024.2 [50]. Reference databases varied according to the target marker: a custom database derived from NCBI entries for *CytB* and the SILVA v138 database for 16S (V4 region) rRNA [53,54]. Unassigned ASVs cross-checked using the NCBI database [March 2025]. In the case of ASVs from libraries of *CytB*, which were assigned to contaminants, non-marine Animalia were removed and excluded from further analysis. These detections included humans (*Homo sapiens*), dogs (*Canis lupus*), pigs (*Sus scrofa*), and cattle (*Bos*), in addition to groups such as insects (class Insecta) and birds (class Aves). Taxonomic relative abundances were visualized as bar plots created in Adobe Illustrator 2025 v.29.8.2. The microbial and ichthyofaunal normalized ASV tables are provided in Supplementary Tables S7 and S8, respectively.

Alpha diversity metrics were calculated based on the normalized ASV table in QIIME 2. Shannon’s entropy diversity index [55], Simpson diversity [56], Margalef richness [57], and Chao1 richness [58]—which accounts for both species richness and abundance—and Pielou’s evenness index [59]—which assesses community evenness—were used to compare samples collected during four different periods (May, June, July, and August). Shapiro–Wilk tests were performed for normality. The results indicated that several indices deviated significantly from normality: Faith’s PD (*p* = 0.0124), Fisher’s α (*p* = 0.0424), Simpson (*p* = 0.000006), and Chao1 (*p* = 0.0018). For Shannon entropy, while the overall distribution was not significantly different from normality (*p* = 0.569), group-level tests revealed deviations in specific cases (e.g., June: *p* = 0.017; August: *p* = 0.038; Station R3: *p* = 0.030). Given these results, nonparametric tests were performed. Diversity values were compared across sampling months, depths (surface vs. thermocline), and stations using the Kruskal–Wallis test and Mann–Whitney U (additionally for the Depth). For pairwise comparisons, Dunn’s post hoc test with Benjamini–Hochberg correction was applied.

Beta diversity was evaluated using Bray–Curtis [60] matrices after applying a log(x + 1) transformation to reduce the impact of dominant taxa while preserving relative relationships. Bray–Curtis dissimilarity was calculated from the transformed ASV table, and the NMDS ordination was computed using the *metaMDS* function (vegan package v.2.7-1 [61]) with two dimensions (k = 2). Environmental variables and ASVs were fitted as vectors onto the NMDS ordination using the *envfit* function (vegan package v.2.7-1 [61]) with 999 permutations to assess the significance of each vector. Community dissimilarities were tested using a factorial PERMANOVA (Bray–Curtis similarity on square-root transformed data) with three fixed factors: Depth (2 levels), Sampling Month (4 levels), and Station (4 levels), including all two-way and three-way interactions, based on 999 permutations of residuals under a reduced model. In addition, a one-way ANOSIM (Analysis of Similarities) was performed separately for the factors of Month, Depth, and Station, using 999 permutations, to provide a complementary rank-based assessment of temporal differences in community structure.

Finally, distance-based redundancy analysis (db-RDA) was selected instead of Canonical Correspondence Analysis-CCA based on the results of Detrended Correspondence Analysis-DCA test (microbial first axis 1.46 SD units and ichthyofaunal 1.08 SD units). Thus, db-RDA was conducted using the capscale function (vegan package v.2.7-1 [61]) in R to explore the influence of environmental parameters on microbial and ichthyofauna community composition, complemented by environmental vector fitting (*envfit*) to identify significant explanatory variables. Relative abundance data of fish ASVs were log-transformed log(x + 1) to reduce the influence of highly abundant taxa while preserving community structure. The data were further Hellinger-transformed to conform with the Euclidean space assumptions of db-RDA. A Bray–Curtis dissimilarity matrix was computed using the *vegdist* function (vegan package v.2.7-1 [61]). The db-RDA was conducted via the *capscale* function with a full model including multiple environmental parameters: pH, dissolved oxygen (concentration and saturation), pressure, temperature, salinity, conductivity, and density (σ). The overall model significance, individual axis significance, and the contribution of each environmental variable were assessed using permutation-based ANOVA tests (999 permutations, *anova.cca*). Multicollinearity among environmental predictors was employed to identify a parsimonious subset of environmental variables that explained significant variation in community composition and was assessed using the *vif.cca* function (*vegan* package v.2.7-1), considering values >10 as indicative of problematic collinearity. Several variables initially showed very high VIF values, confirming redundancy. To address this, we applied a stepwise selection procedure (*ordistep*) (vegan package v.2.7-1 [61]), starting from a null model and using the full model as scope. This approach iteratively removed redundant predictors and retained only those variables that significantly improved model fit, resulting in the final reduced db-RDA model. Partial db-RDA was used to control for potential confounding variables when necessary, enabling assessment of the unique contribution of selected environmental factors. Ordination plots were generated with *ggplot2* to visualize the relationship between samples and constraining variables. Biplot arrows indicated the direction and strength of environmental gradients. Additionally, the variance explained by each variable was visualized using barplots, with F-values and *p*-values annotated. Adjusted R^2^ values were computed to evaluate model fit.

### 2.6. Comparative Analysis of Microbiome and Ichthyofauna

A series of multivariate and network-based analyses was employed in R to investigate the relationships between microbial and ichthyofaunal communities and environmental variables in coastal ecosystems. Procrustes analysis was performed using non-metric multidimensional scaling (NMDS) ordinations based on Bray–Curtis dissimilarities. Rarefied ASV tables for both microbiome and ichthyofaunal communities were standardized using the Hellinger transformation to account for compositional bias and reduce the impact of rare taxa. Two-dimensional NMDS ordinations were independently computed for each community using the *metaMDS* function from the vegan R package v.2.7-1 [61]. The Procrustes rotation (*protest*) (vegan package v.2.7-1 [61]) was then applied to assess the spatial concordance between the ordinations. The resulting Procrustes correlation statistic (r) and permutation-based *p*-value (999 permutations) were extracted to quantify the strength and significance of the alignment. Visualization of the Procrustes transformation was achieved using ggplot2, with arrows depicting the translation of points (samples) from one configuration to the other. The Mantel statistic (Pearson’s r) and associated *p*-value (999 permutations) were computed using the *mantel* function (vegan package v.2.7-1 [61]).

Finally, co-occurrence network analysis was conducted separately for surface and thermocline layers. ASV tables for bacteria and fish, along with environmental metadata, were exploited. Samples were grouped by depth. Taxa present in at least 5% of samples were selected to reduce noise. Pairwise Spearman correlations were calculated between all taxa, and significant associations were defined by |ρ| ≥ 0.6 and *p* < 0.05. Networks were built using the *igraph* package v.2.2.0.9002, with nodes representing taxa and edges representing significant correlations. Topological metrics such as degree, betweenness, and community structure (Louvain clustering) were computed. Each taxon was annotated with a trophic strategy (autotroph, heterotroph, oligotroph, mixotroph, predator) based on literature-inferred classifications. Networks were visualized with *ggraph* [62] using force-directed layouts. To assess the structural properties of microbial–eukaryotic co-occurrence networks, a range of graph-theoretical metrics was calculated for both surface and thermocline networks. Metrics included network size (number of nodes and edges), density, average node degree, average shortest path length, and network diameter. Additionally, centrality measures such as mean closeness and betweenness centrality were computed to evaluate the relative influence of nodes within the network. The global clustering coefficient was used to estimate the tendency of taxa to form tightly connected clusters. All metrics were calculated using the igraph R package v.2.2.0.9002 and network connectedness was confirmed using *is_connected* [63].

## 3. Results

### 3.1. Environmental Variability

**Figure 2:**
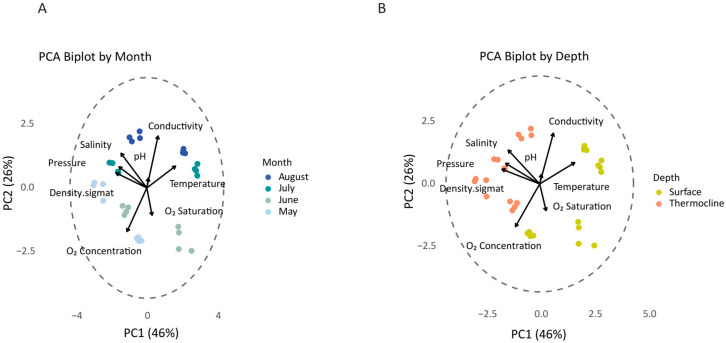
Multivariate analysis of environmental variables of seawater samples collected from the study area between May and August 2024. (**A**) Principal Component Analysis (PCA) based on Euclidean distance revealed a clear temporal separation of samples based on the environmental variables, highlighting seasonal environmental shifts. (**B**) PCA analysis colored by depth demonstrated distinct surface and thermocline sample clustering, indicating a strong influence of the vertical stratification on the environmental parameters.

A total of 96 seawater samples were collected during the period of study (May to August, S1–S4, Figure 1) at two different water depths, which correspond to two water layers, surface (A) and thermocline (B), from four sampling stations (Locations R1–R4). To characterize the seawater conditions during sampling, environmental variables were monitored (Supplementary Table S1). Environmental variables showed distinct seasonal and depth-related patterns throughout the sampling period (Supplementary Figure S1A–G, Table S1). Surface water temperatures exhibited a progressive increase from May (average ~18 °C) to a peak in July and August (~27 °C), consistent with expected seasonal warming trends. Conductivity followed a parallel increase, reaching maximum values in August (over 52 mS/cm). Salinity values oscillated between 31 and 39 PSU, with relatively lower measurements recorded in June and higher in July and August. The pH remained relatively stable across the months (8.5–8.8). The water density mirrored changes in temperature and salinity, decreasing during June before recovering by August. Oxygen saturation displayed a seasonal peak in June (~107%), with values declining towards August (~85–90%), corresponding to increased water temperature and stratification. Similarly, oxygen concentrations (mg/L) decreased from May to August, suggesting reduced oxygen solubility due to warming and decreased photosynthetic activity as a consequence of nutrient depletion that typically occurs during the late summer period. Principal Component Analysis (PCA) indicates clear clustering by sampling month and depth, highlighting the seasonal and vertical structure of the physicochemical environment in the studied area (Figure 2A,B).

### 3.2. Temporal and Depth Affect the Microbiome Diversity

**Figure 3:**
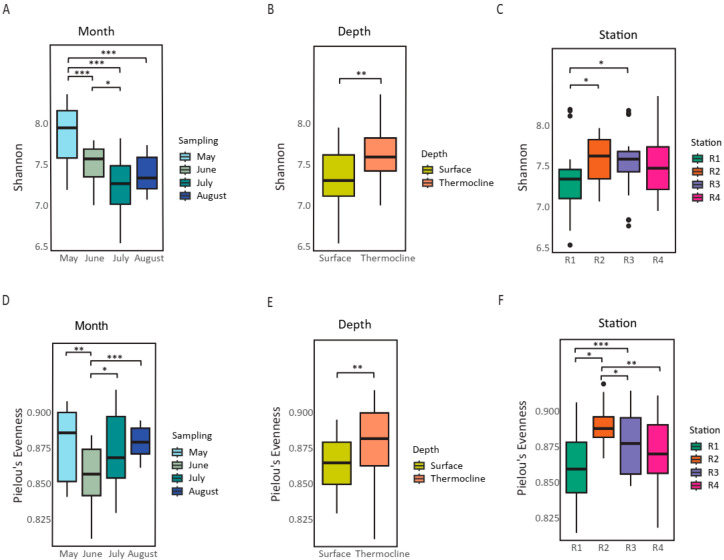
Alpha diversity comparisons of the microbial community across temporal, vertical, and spatial scales. (**A**–**C**) Boxplots showing variation in Shannon entropy across (**A**) Temporal (Monthly) sampling (May, June, July, August), (**B**) Depth (surface vs. thermocline), and (**C**) Stations (R1–R4). (**D**–**F**) Boxplots showing variation in Pielou’s evenness across (**D**) Temporal (Monthly) sampling, (**E**) Depth, and (**F**) Stations. Statistical significance was assessed using the Kruskal–Wallis test and Dunn’s post hoc test, with asterisks denoting levels of significance (* *p* < 0.05, ** *p* < 0.01, *** *p* < 0.001; Supplementary Table S3). Significant seasonal and spatial differences were observed in alpha diversity metrics, highlighting the influence of temporal, vertical, and spatial factors on microbial community structure.

Following the characterization of physicochemical parameters, microbial diversity patterns were examined. In total, 6,949,640 sequencing reads were generated for microbiome analysis (i.e., about 40,500 per sample). Sequences were assigned to a total of 4595 ASVs. Alpha diversity indices displayed clear seasonal and depth-related patterns across the sampling months (May–August). Shannon entropy, Margalef richness, and Chao1 estimator richness all showed significantly higher values during May, followed by a marked decrease toward July and August (Kruskal-Wallis, *p* < 0.01 for all indices; Supplementary Figure S2A–E, Figure 3A,D, Supplementary Table S2). Post hoc comparisons (Dunn’s test with Benjamini–Hochberg correction) revealed that May samples differed significantly from June, July, and August, particularly for Shannon indices (Kruskal-Wallis, *p* = 4.3 × 10^−7^, Figure 3A,D, Supplementary Table S2 and S3). Thermocline samples consistently exhibited higher diversity and richness compared to surface samples (Figure 3B,E, Supplementary Tables S2 and S3), with significant differences observed for Shannon entropy (Kruskal–Wallis, *p* = 0.000528; and Mann–Whitney U test U statistic = 679.0 *p* = 0.00054) and Pielou’s evenness (Kruskal-Wallis, *p* = 0.00479; Mann–Whitney U, *p* = 0.004840), suggesting enhanced stratification effects with depth. Margalef and Chao1 estimators highlighted greater taxonomic richness at the thermocline, especially early in the season, while Simpson presented less variance in richness between the groups (Supplementary Figure S2, Supplementary Table S2). Station-specific variation was rather minor and less pronounced than temporal and depth-related trends (Figure 3C,F). Overall, alpha diversity metrics collectively demonstrated a seasonal decline in richness and evenness, likely driven by the progression of thermal stratification, possible reduced nutrient availability, and potential shifts in microbial community structure.

**Figure 4:**
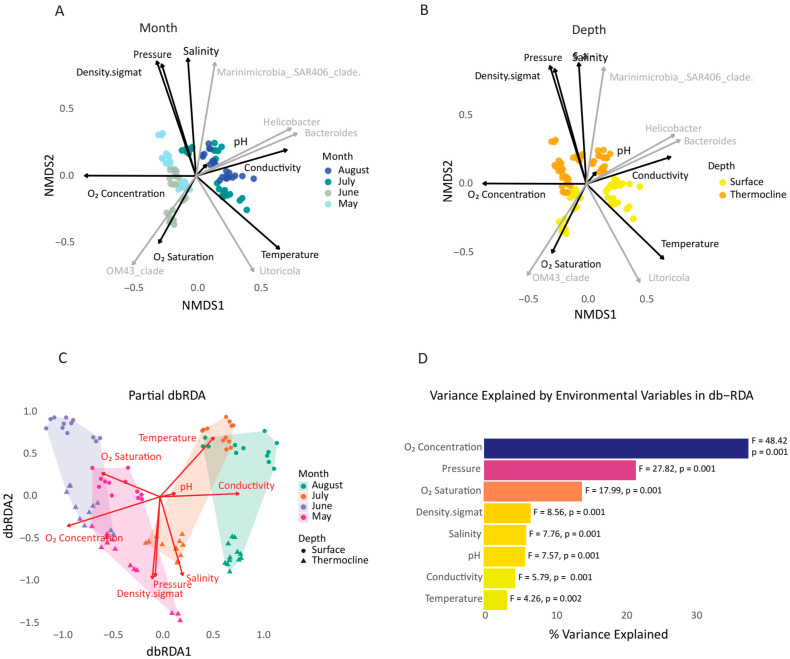
Structure of microbial community based on Bray–Curtis similarity and environmental variables. NMDS of microbial beta diversity patterns based on Bray–Curtis similarity. (**A**) Samples are colored by Month (May, June, July, August). Distinct temporal clustering is observed, highlighting strong seasonal transitions in microbial community composition. (**B**) Samples are colored by Depth (surface vs. thermocline layer). Clear vertical separation between communities reflects the role of water column stratification. The lengths and directions of the fitted vectors represent the strength and direction of correlation with the ordination configuration of environmental parameters. The top five ASVs with the longest vector lengths were also identified and labeled. Partial distance-based redundancy analysis (partial db-RDA) on microbial community composition based on environmental variables. (**C**) db-RDA ordination plot showing sample distribution by month (May to August, color-coded) and depth layer (surface vs. thermocline, indicated by point shape). Red vectors represent the environmental variables significantly contributing to community variation. (**D**) Bar plot illustrating the proportion of variance explained (F-values) by each environmental variable. O_2_ Concentration and Pressure explain the largest portion of the observed variation.

The Bray–Curtis similarity compares the relative abundances and quantifies the difference in composition between samples. ANOSIM test (Analysis of similarities) is a rank-based non-parametric method relying on Bray–Curtis similarities to detect patterns by comparing within- and between-group similarities under permutation testing (higher R values indicate strong separation between groups, while lower R values suggest partial overlap). Beta diversity patterns based on Bray–Curtis similarity were strongly structured by temporal, vertical, and spatial factors (NMDS; Figure 4A, B, Supplementary Figure S3). Moreover, the microbial community composition differed significantly over time/month (PERMANOVA analysis Pseudo-F = 85.49, *p* = 0.001, ANOSIM analysis Global R = 0.642, *p* = 0.001). Interestingly, analysis revealed pronounced temporal variation in microbial assemblages, particularly separating May and June from July and August (Figure 4A). This pattern suggests a strong seasonal succession likely associated with temperature-driven stratification and nutrient dynamics across the summer. Depth was also a major factor structuring microbial assemblages (PERMANOVA analysis Pseudo-F = 103.71, *p* = 0.001, ANOSIM analysis Global R = 0.372, *p* = 0.001), highlighting the influence of water column layering on bacterial assemblages. Surface and thermocline communities showed clear separation in the ordination space (Figure 4B), supporting the role of vertical gradients (e.g., light, oxygen, nutrient concentrations, etc.) in shaping microbial distributions. The effects of depth were consistently significant throughout the sampling month and were further supported by interaction terms (Depth × Month and Depth × Station). In contrast, although the sampling station had a statistically significant contribution, its effect was responsible for a comparatively smaller portion of the total observed variance (PERMANOVA analysis Pseudo-F = 12.06, *p* = 0.001, ANOSIM analysis R = 0.028, *p* = 0.066), indicating rather subtle compositional differences between stations. Spatial structuring among stations was weaker than the temporal or depth patterns, although significant differences were still detected, possibly reflecting small local environmental heterogeneities (Supplementary Figure S3). In aggregate, the analysis underlines the dominant role of seasonal progression and vertical water column stratification in structuring microbial beta diversity patterns in the study region.

To quantify the influence of environmental gradients on microbial community composition, a partial distance-based redundancy analysis (partial db-RDA) was performed using Bray–Curtis dissimilarities. The ordination captured clear structuring of microbial assemblages along environmental axes, with strong separation by sampling month and depth (Figure 4C). Surface and thermocline samples, denoted by different point shapes, exhibited distinct clustering patterns. Samples from July and August (green and orange) were positioned along gradients associated with increased temperature and conductivity, while samples from May and June (blue and pink) were strongly associated with higher oxygen concentration and oxygen saturation, particularly in deeper layers.

The overall model was highly significant (F = 15.56, *p* = 0.001), explaining 3.225 units of variance out of 5.414 total inertia, corresponding to approximately 59.57% of the total variation (Figure 4D). All environmental predictors included in the model exhibited a statistically significant effect (*p* < 0.001), with O_2_ concentration, pressure, and O_2_ saturation contributing the most to community structure, as indicated by their high F-values (F = 48.42 for O_2_ concentration). Though the first two constrained axes (CAP1 and CAP2) captured the majority of the explained variance (23.2% and 17%, respectively), even CAP8 remained statistically significant (*p* < 0.05). A permutation test on marginal effects (*n* = 999) showed that all of the environmental variables (including pH, oxygen concentration and saturation, pressure, temperature, salinity, conductivity, and density) significantly explained microbial beta diversity (all *p*-values = 0.001). The ordination biplot revealed a clear separation of samples across sampling months and depths. Summer samples were associated with higher temperature and conductivity, while deeper samples aligned with elevated oxygen and pressure. Overall, the db-RDA results highlight that microbial communities in the Thracian Sea are structured by a combination of oxygen availability, hydrostatic pressure, and thermal stratification-related parameters, suggesting that oxygen dynamics and water column structure are key environmental drivers shaping bacterial assemblages during summer.

### 3.3. Microbial Community Structure and Core

**Figure 5:**
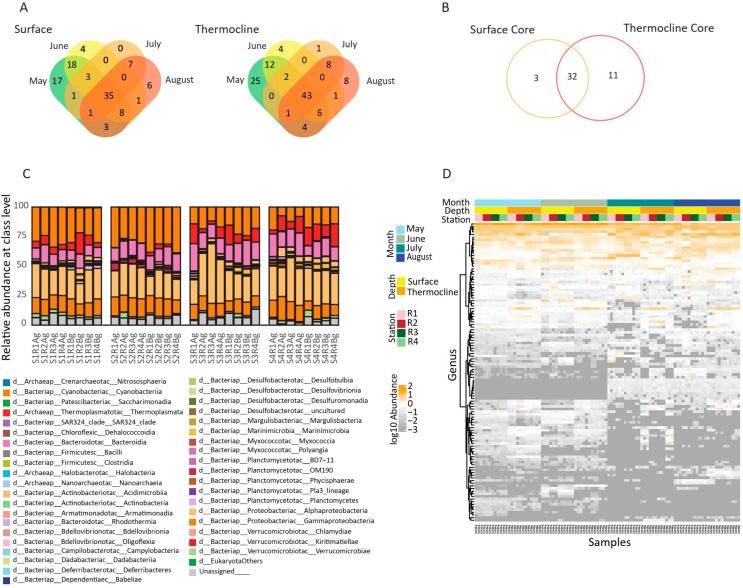
Microbial community composition and core microbiome analysis across spatial and temporal gradients in the Thracian Sea. (**A**) Venn diagrams showing the shared and unique species across sampling months (May–August) for surface samples. Equivalent Venn diagram for thermocline samples, with overlapping species among all months defining the thermocline core microbiome. (**B**) Venn diagram showing the overlap between the core species identified in the surface and thermocline layers. Shared ASVs (*n* = 32) represent the core microbiome of the entire water column. (**C**) Relative abundance bar plot of dominant microbial classes across all samples, grouped by depth and secondary month. (**D**) Heatmap representing the relative abundance of the top 100 most abundant genera across samples. Hierarchical clustering of genera (rows) reveals patterns of co-occurrence and seasonal/vertical structuring. The color gradient reflects scaled abundance, with orange indicating higher relative abundance and dark grey indicating lower relative abundance. This integrative figure summarizes the seasonal dynamics, taxonomic structure, and persistent microbial signatures across the coastal microbial community in the Thracian Sea.

To identify the core microbiome across depths and sampling periods, a stepwise filtering strategy was applied. Initially, for each biological replicate (triplicate samples) only taxa present in at least two out of the three replicates were retained, ensuring robustness against stochastic sequencing noise. Subsequently, we defined the surface or thermocline core microbiome detected in each month. For each sampling month, taxa consistently detected in all four surface sampling stations were considered members of the surface-associated core microbiome (Supplementary Figure S4), whereas those consistently found in all four thermocline stations formed the thermocline-associated core (Figure 5A). This approach revealed a dynamic yet distinct core assemblage at each depth, with 35 and 43 taxa identified as core members in the surface and thermocline samples, respectively, throughout the studied period. Subsequently, a comparative analysis between the two depth-specific cores showed that 32 taxa were shared between surface and thermocline layers, constituting the general core microbiome across the coastal water column during the study period (Figure 5B).

To explore the structure and temporal dynamics of the microbial communities in the study area, we assessed the relative abundance of bacterial taxa at the class levels across all stations, sampling months, and depths (Figure 5C). The microbial assemblages were consistently dominated by a limited number of bacterial classes, with notable variation in their relative contributions across space and time. At the class level, Alphaproteobacteria, primarily the SAR11 clade, emerged as the most dominant group across all samples, consistently representing a substantial portion of the community throughout the study period. This was followed by Gammaproteobacteria, Bacteroidia, and members of Planctomycetota and Verrucomicrobiota, which also displayed considerable relative abundance. The SAR11 clade showed clear seasonal variation, peaking during late spring and early summer, especially in surface waters. Cyanobacteria (*Prochlorococcus* and *Synechococcus*) also exhibited seasonal peaks, becoming more prominent during the months of May and June. Notably, *Prochlorococcus* exhibited higher relative abundance at the thermocline or deeper depths, suggesting niche specialization. Also, classes affiliated with Planctomycetes and Marinimicrobia exhibited higher relative abundance at the thermocline, suggesting possible responses to environmental gradients such as oxygen concentration and temperature stratification.

Conversely, a large number of ASVs were classified as rare or conditionally rare, appearing only sporadically or in low abundance. The genus-level heatmap (Figure 5D) further highlighted shifts in dominant taxa across samples. While a few genera, especially those affiliated with SAR11 and SAR86, Flavobacteriales, and Rhodobacteraceae, maintained a consistent presence, other taxa exhibited strong spatiotemporal variability. For example, taxa such as Lentimonas and OM60 (NOR5) clade were more abundant during early summer, while AEGEAN-169, Clade_Ia, Marinomicrobia (SAR406_clade), and Rickettsiales (S25-593) appeared to peak in mid- to late-summer. In summary, the microbial communities exhibited clear vertical and temporal structuring, with strong dominance of oligotrophic (SAR groups and Thermoplasmata) and phototrophic taxa (Cyanobacteria) in surface layers and a broader taxonomic diversity, including heterotrophic and uncultured lineages, in deeper or thermocline-associated samples. These results suggest a dynamic microbial ecosystem shaped by temporal shifts and stratification, with a stable core community coexisting with a large fraction of transient and rare taxa.

### 3.4. Temporal Effects Dominate over Spatial Variation in Ichthyofaunal Diversity

To broaden our understanding of ecosystem functioning, we delved into marine life biodiversity data. While microorganisms govern essential biogeochemical processes, fish communities reflect higher trophic-level responses and integrate longer-term environmental signals. In total, 9,163,332 reads from the *mtCytB* genetic marker were generated, with an average of 53,500 reads per sample. Sequences were assigned to a total of 5663 ASVs. After filtering out the reads that do not correspond to marine life, the analysis of eDNA data, assigned to 30 species, revealed temporal structuring of fish communities across the four sampling months in the Thracian Sea. Alpha diversity indices (Shannon, Pielou’s Evenness) showed no statistically significant differences across sampling months, depths, or stations (Supplementary Figure S5). The same lack of statistically significant behaviour was present in Margalef and Simpson. The absence of pronounced alpha diversity segregation likely reflects the small geographical scale of our study area combined with the high dispersal capacity of marine fish species and likely reflects the limitations of eDNA for quantitative community assessment in fish, especially at the species resolution level.

**Figure 6:**
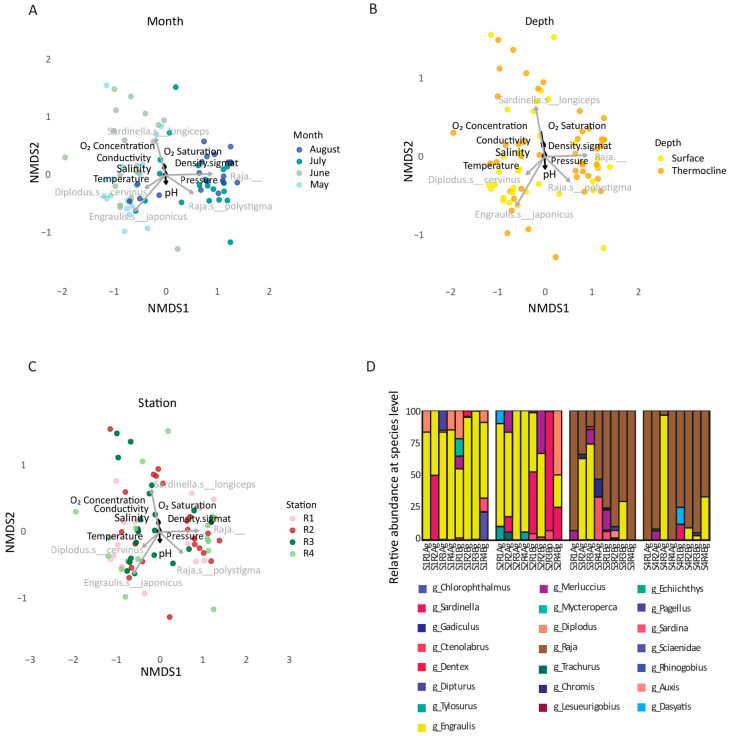
Spatiotemporal patterns and taxonomic composition of fish communities in the Thracian Sea. NMDS plots based on Bray–Curtis dissimilarities of marine life community composition, showing clustering patterns by (**A**) sampling month, (**B**) depth (surface vs. thermocline), and (**C**) station (R1–R4). The lengths and directions of the fitted vectors represent the strength and direction of correlation with the ordination configuration of environmental parameters. The top five ASVs with the longest vector lengths were also identified and labeled. Distinct grouping by sampling month is evident, while depth and station-level clustering are less pronounced. (**D**) Bar plot of relative abundance (%) of marine species detected across all samples, grouped by depth and month. Each color represents a different fish species, highlighting temporal-specific variability in community composition.

Fish communities exhibited temporal variation across the sampling period from May to August. Non-metric Multidimensional Scaling (NMDS) plots based on Bray–Curtis dissimilarities showed a distinct clustering of samples by month (Figure 6A). This suggests that seasonal turnover played a major role in shaping fish community composition, with May and June samples generally clustering apart from those in July and August. When considering the depth (Figure 6B), we observed that separation was less pronounced than seasonal effects. This implies that depth (surface and thermocline layer) is not the dominant factor in fish community structuring. Similarly, the stations (Figure 6C) revealed no consistent spatial segregation among the four sampled locations, indicating that spatial variation across stations was relatively minor compared to temporal dynamics.

Focusing on the taxonomic composition at the genus level, the genus *Raja* dominated a large portion of the samples, especially in July and August, suggesting possible seasonal abundance, shedding, or reproduction patterns (Figure 6D). Among the taxa included were *Sardina pilchardus*, *Engraulis encrasicolus*, *Diplodus sargus*, and *Pagellus erythrinus*, which were intermittently detected across months and locations (Supplementary Table S4). In addition, eDNA analysis revealed the sporadic presence of a wide range of teleost and elasmobranch species, including taxa not typically endemic to the study area, such as *Engraulis japonicus*, *Trachurus lathami*, *Merluccius albidus*, and *Sardinella longiceps*, as well as *Gadiculus argenteus*, a species known from the region but generally inhabiting much deeper waters. These detections may partly reflect taxonomic misannotations in reference databases combined with the limited genetic variability of the selected genetic marker, which can hinder accurate species-level discrimination, but they contributed to the observed richness, albeit often at low relative abundance. Overall, these patterns highlight a dynamic and seasonally structured ichthyofaunal community, with notable turnover in detected taxa over time and limited spatial segregation. The results also suggest that surface and thermocline samples captured overlapping, but not identical, community compositions.

**Figure 7:**
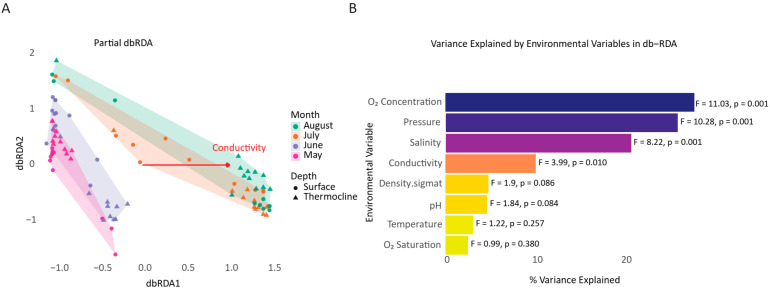
Distance-based redundancy analysis (db-RDA) of ichthyofaunal diversity patterns based on Bray–Curtis similarity. (**A**) Partial distance-based redundancy analysis (partial db-RDA) ordination plot showing ichthyofaunal community structure constrained by environmental variables. Samples are grouped by month (May to August) and depth (surface: circles, thermocline: triangles). Shaded ellipses represent 95% confidence intervals per sampling month. The red vector represents the most influential environmental gradient on community composition along the canonical axes. (**B**) Bar plot showing the percentage of variance in microbial community composition explained by each environmental variable, based on marginal effect tests (permutation test, *n* = 999). Oxygen concentration and pressure explained the largest share of variation (F = 11.03 and F = 10.28, respectively), followed by salinity (F = 8.22).

The partial db-RDA model revealed that environmental parameters explained a limited yet statistically significant proportion of variation in fish community composition (Figure 7). The total constrained inertia accounted for 22.75% of the variation (7244 out of 31,841 total inertia). Among the tested variables, O_2_ Concentration (F = 11.03, *p* = 0.001), pressure (F = 10.28, *p* = 0.001), salinity (F = 8.22, *p* = 0.001), and conductivity (F = 3.99, *p* = 0.007) exhibited a statistically significant effect, indicating a strong association between fish assemblage structure and these environmental parameters’ gradient across sampling sites. These results highlight O_2_ concentration, pressure (depth), and salinity as possible environmental drivers influencing ichthyofaunal distribution during the study period, potentially reflecting their correlation with salinity gradients, stratification, or water mass origins. Nevertheless, most of the variation in fish community structure (77.25%) remained unexplained by the measured abiotic parameters, implying a prominent role of biological, spatial, or stochastic factors, such as species-specific behavior, life cycles, dispersal patterns, or unmeasured influences like prevailing marine currents and fishing activity that are not directly captured by environmental DNA concentrations alone.

### 3.5. Linking Microbial and Fish Biodiversity in Coastal Ecosystems

A series of multivariate and network-based analyses were employed to investigate the relationships between microbial communities, ichthyofaunal assemblages, and environmental variables in coastal ecosystems. Preliminary comparisons suggest congruent temporal trends between microbial diversity indices and fish species richness, with both showing declines during peak summer conditions. These parallel patterns may reflect shared responses to environmental gradients. Further analyses investigate potential correlations between microbial community structure and fish assemblage metrics, aiming to explore potential ecological links of biodiversity in this dynamic coastal system.

**Figure 8:**
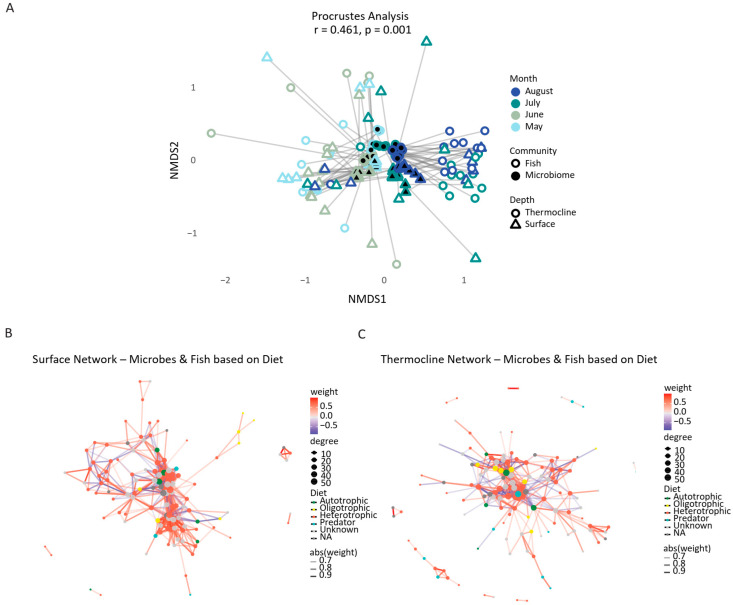
(**A**) Procrustes analysis comparing microbial (black fill) and fish (white fill) community structures across sampling sites. Each line connects corresponding microbial and fish communities at the same sample, representing the degree of concordance between the two datasets in NMDS space. The analysis revealed a moderate but statistically significant association (Procrustes correlation r = 0.461, *p* = 0.001), suggesting that microbial and ichthyofaunal assemblages share underlying spatial or environmental structuring. (**B**,**C**) Co-occurrence network analysis of microbial and eukaryotic taxa across depth layers. (**B**) Surface layer. (**C**) Thermocline layer. Nodes represent individual ASVs or taxa, colored by ecological or taxonomic identity: green for autotrophic microorganisms (e.g., Cyanobacteria, algae), yellow for oligotrophs, red for heterotrophic microbial taxa, and blue for higher eukaryotes/predators (e.g., fish). Edges represent significant Spearman correlations (|R| > 0.6, *p* < 0.05): red for positive correlations and blue for negative. Edge width is proportional to correlation strength (R).

To assess the degree of alignment between microbial and fish community structures, a Procrustes analysis was conducted on NMDS ordinations derived from Bray–Curtis dissimilarity matrices. The analysis revealed a moderate but statistically significant concordance between the two datasets (Procrustes correlation r = 0.461, *p* = 0.001), indicating that microbial and ichthyofaunal assemblages shared underlying spatial or temporal patterns (Figure 8A). Despite differences in ecological scale and mobility between microbes and fish, the observed alignment suggests that both community types may be structured, at least in part, by common environmental patterns with respect to spatial and temporal variability. Mantel statistical analysis confirmed the existence of a significant, albeit moderate, correlation between the distances of fish and microbial communities (r = 0.214, *p* = 0.001), suggesting that the two biotic components of the marine ecosystem are ecologically co-shaped. These results support the existence of coordinated biodiversity responses to environmental variation. Together, the Procrustes and db-RDA results highlight a spatial and temporal structuring of microbial and ichthyofauna communities, governed by a set of shared abiotic factors.

Potential ecological associations between microbial and eukaryotic taxa across different water depths were explored by performing co-occurrence network analysis. Co-occurrence network analysis revealed distinct structural and ecological patterns between the surface and thermocline layers. The comparison of co-occurrence network metrics between surface and thermocline layers verified distinct structural properties (Figure 8B, C, Supplementary Table S5). The surface network exhibited a larger size and higher connectivity, with 179 nodes and 629 edges compared to 135 nodes and 309 edges in the thermocline network. This was further supported by the increased average degree (7.03 vs. 4.58) and clustering coefficient (0.41 vs. 0.35), suggesting more frequent and tighter associations among taxa at the surface. Notably, mean betweenness centrality was also higher in the surface layer (72.1), highlighting the presence of key taxa acting as central connectors in the network. Despite these differences, both networks were fully connected, indicating cohesive ecological structures at both depths. Possibly, these patterns collectively underscore a shift from a phototroph-dominated, cooperative community at the surface to a more functionally specialized, modular, and potentially competitive microbial ecosystem at depth.

## 4. Discussion

The comprehensive spatiotemporal analysis of microbial and ichthyofaunal communities in the Thracian Sea has revealed consistent patterns of seasonal structuring, vertical differentiation, and moderate inter-taxa concordance. These findings provide a detailed characterization of biodiversity dynamics in a semi-enclosed marine ecosystem during a period of intense thermal stratification. To place these observations within a broader ecological and methodological context, we should consider the detected patterns, the ecological significance of the microbial core and fish community composition, and the potential limitations of eDNA-based monitoring for integrated biodiversity assessments in coastal environments.

### 4.1. Seasonality and Depth as Principal Axes of Microbial Variation and Microbial Core

Our results indicate that thermal stratification during summer months leads to a progressive decline in microbial diversity from May through August in the Thracian Sea. This trend aligns with the “stratification-induced oligotrophy” model, where nutrient limitation due to limited vertical mixing results in reduced microbial richness. Similar seasonal and depth-driven structuring of microbial communities has been observed across the Mediterranean, where the most microbial taxa in stratified Mediterranean waters exhibit narrow vertical niches, with only a few taxa such as SAR11 showing depth ubiquity, and strong seasonal stratification driving functional and taxonomic differentiation at fine scales [64]. Similarly, the apparent richness in deep bacterial communities increases as stratification decreases, pointing to the homogenizing effect of winter mixing [65]. These patterns resonate with other observations, where microbial communities in the Mediterranean and Aegean Seas also reflected strong seasonal shifts modulated by thermal layers and nutrient gradients [17,18].

Despite marked seasonal and vertical shifts in microbial composition, several taxa persist across temporal gradients, forming a stable “core microbiome” in the Thracian Sea. The microbial core consists primarily of taxa consistently associated with oligotrophic marine systems. It includes dominant representatives of Alphaproteobacteria (particularly multiple SAR11 clades), Bacteroidota (notably marine Flavobacteriales groups like NS4, NS5, NS7, and NS9), Cyanobacteria (e.g., *Synechococcus*), Planctomycetes, Actinobacteriota, Marinimicrobia (SAR406), Verrucomicrobiota, and Archaea (Thermoplasmata). This taxonomic composition points to a core adapted to low-nutrient, stable open-sea environments, and capable of metabolizing a wide range of dissolved organic matter. SAR11 clade (Clades I, II, III, Ia, Ib) dominates, suggesting streamlined genome ecotypes optimized for carbon cycling in nutrient-poor conditions [66,67]. Flavobacteriales (NS4, NS5, NS7, NS9), known for their role in degrading high molecular weight organic matter, suggest a specialization for the late stages of phytoplankton bloom recycling [68]. *Synechococcus* represents a photoautotrophic component, vital for primary production and linked to nitrogen fixation or DOM (Dissolved Organic Matter) excretion [69]. Thermoplasmata (Marine Group II Archaea) have been repeatedly associated with protein and lipid degradation and are metabolically active in surface and deep waters [15]. Together, these taxa form a multi-functional core. SAR11 and Thermoplasmata dominate carbon oxidation and nutrient cycling. Flavobacteriales contribute to DOM remineralization. Cyanobacteria (e.g., *Synechococcus*) lineages support primary productivity. Planctomycetes, Verrucomicrobia, and Marinimicrobia offer specialized degradative functions and symbiotic potential. This microbial core appears to be conserved across seasons and depth strata, providing ecosystem stability and acting as a bioindicator of oligotrophic resilience, in accordance with previous observations [64].

### 4.2. Ichthyofaunal Diversity in the Thracian Sea

The environmental DNA (eDNA) analysis revealed a diverse assemblage of teleost and chondrichthyan fish in the Thracian Sea, spanning multiple trophic and ecological guilds. Dominant representatives included small pelagic species of commercial importance such as *Engraulis encrasicolus* (European anchovy), *Sardina pilchardus* (sardine), and *Trachurus* sp. (horse mackerel), all key species in local fisheries and the eastern Mediterranean food web. Members of the Sparidae family, including *Diplodus sargus*, *Pagellus erythrinus*, and *Dentex gibbosus*, were also frequently detected, indicating a rich demersal component and potential coastal affinity. Apex or higher trophic level taxa were represented by species such as *Mycteroperca* sp. and *Auxis* sp., whereas elasmobranchs such as *Dasyatis tortonesei*, *Raja miraletus*, and *Dipturus oxyrinchus* point to the presence of sensitive or vulnerable cartilaginous species.

This taxonomic breadth suggests that eDNA sampling captured both neritic and benthopelagic species. The co-detection of species from different habitat zones (e.g., *Lesueurigobius friesii* in coastal soft substrates and *Merluccius* sp. from deeper continental shelf areas) supports the utility of eDNA in integrative ichthyofaunal monitoring. Notably, several of the unexpected fish species assignments (e.g., *Mycteroperca bonaci*, *Engraulis japonicus*, *Trachurus lathami*, *Sardinella longiceps*, *Chromis scotti*) likely reflect database-related misannotations rather than true occurrences in the Thracian Sea. Misassignments in CytB-based eDNA metabarcoding can arise due to (i) high genetic similarity among closely related species, (ii) incomplete representation of Mediterranean taxa in public repositories, (iii) erroneous or outdated database entries, and (iv) limited resolution of *CytB* for certain taxonomic groups [70,71]. Indeed, large-scale evaluations of *CytB* sequences in GenBank have documented high rates of misannotations and unreliable entries [72].

Other fish species detected in our study using eDNA, including *Sardina pilchardus*, *Engraulis encrasicolus*, *Trachurus* sp., and several *Sparidae,* are broadly consistent with those commonly reported in Mediterranean surveys. For example, in pelagic waters off Lebanon, *Clupeidae* and *Engraulidae* comprised over 90% of the abundance in juvenile fish assemblages, with species such as *Sardina pilchardus*, *Scomber japonicus*, and *Engraulis encrasicolus* dominating seasonal catches [73]. Similar trends were observed in the northern Lebanese coast, where eDNA and purse seine sampling identified strong seasonal turnover between these same taxa [74]. Additionally, Sparidae species such as *Diplodus sargus*, *Pagellus erythrinus*, and *Dentex* spp. have been frequently reported in both catch-based studies and molecular assessments across the eastern Mediterranean and Aegean Seas [75].

These alignments between our eDNA-based records and traditional survey data highlight the reliability of molecular tools for monitoring ichthyofaunal diversity in the region. Moreover, they emphasize the ecological consistency of key commercial and ecological species across the eastern Mediterranean basin.

### 4.3. Environmental Drivers of Microbial and Ichthyofaunal Communities

The results of the partial distance-based redundancy analysis (db-RDA) revealed that microbial and fish communities responded differently to the measurable environmental gradients, reflecting distinct ecological sensitivities and mechanisms of structuring.

For the microbial communities, the constrained db-RDA model explained a substantial portion of the total variance (59.7%), highlighting a strong environmental imprint on microbial beta diversity. Among the tested predictors, oxygen concentration, pressure, and oxygen saturation emerged as the most influential drivers. This suggests that oxygen availability and hydrostatic pressure, parameters tightly linked to depth and stratification, are key factors structuring microbial assemblages in the Thracian Sea during summer. The observed separation between surface and thermocline samples in the db-RDA space aligns with known microbial adaptations to oxygen gradients and photic zone limitations. Variables such as salinity, pH, and temperature also contributed meaningfully to microbial variance, albeit to a lesser extent, reflecting the multifactorial nature of environmental filtering in microbial ecology.

In contrast, ichthyofaunal communities exhibited a weaker relationship with environmental gradients. The db-RDA model for fish explained only 22.8% of the total inertia. Among the predictors, O_2_ concentration, pressure, salinity, and conductivity showed a statistically significant association with fish community composition. This may reflect indirect effects of salinity gradients or water mass structure, influencing species occurrence through physiological tolerances or habitat preference.

These contrasting results highlight the higher environmental sensitivity and niche fidelity of microbial communities, which respond rapidly and predictably to physicochemical conditions, compared to fish assemblages, which are influenced by broader spatial processes (e.g., migration, feeding, spawning), life-history traits, and eDNA transport/diffusion dynamics. Additionally, the reduced explanatory power for ichthyofauna may reflect methodological limitations, such as differential eDNA shedding and degradation rates, or challenges in detecting mobile organisms from water samples at a fixed point in time.

Overall, the db-RDA analyses emphasize the value of microbial eDNA as a fine-scale bioindicator of environmental gradients, while fish eDNA signals likely integrate both environmental and ecological factors, requiring careful interpretation.

### 4.4. Microbe–Fish Community Coupling

Our study provides statistical evidence (Mantel, Procrustes, and co-occurrence network analyses) for a correlation between microbial and fish community structures. These results represent hypothetical associations inferred from correlations and not direct biological interactions. However, despite biological and ecological differences, both communities exhibit coordinated temporal structuring.

This type of trophic or functional coupling is increasingly recognized in marine systems. In the Strait of Sicily, eDNA metabarcoding of water samples combined with fishing data enabled the reconstruction of demersal food webs, confirming consistent trophic links between microbes, plankton, and fish across gradients of fishing intensity [76]. Similarly, in Monterey Bay, network analysis based on eDNA time series revealed putative trophic and seasonal co-occurrence relationships across microbial and higher taxa, including fish, further highlighting the systemic connections across trophic levels [77].

## 5. Conclusions

Our integrated analysis of microbial and fish communities in the Thracian Sea reveals clear patterns of seasonal structuring, trophic coupling, and biodiversity consistent with other Mediterranean regions. The detection of a functionally robust microbial core and diverse ichthyofaunal signatures signifies the importance of eDNA application, as a time- and cost-efficient, powerful tool in ecosystem monitoring.

Future work should aim to enhance quantitative resolution and temporal continuity, to fully capture the dynamics of microbial–macrobial interactions in response to climate and anthropogenic pressures. Ultimately, the outcomes will contribute to improved management of marine ecosystems, supporting data-driven decision-making and long-term conservation strategies under the framework of marine spatial planning. They can also support the development of targeted monitoring programs for assessing fish population trends and detecting changes in community structure across seasons.

## Data Availability

The original contributions presented in this study are included in the article/Supplementary Material. Further inquiries can be directed to the corresponding authors.

## Supplementary Materials

The following supporting information can be downloaded at: https://www.mdpi.com/article/10.3390/microorganisms13102373/s1, Supplementary Figure S1: Seasonal variation of physicochemical parameters of seawater samples collected from the study area between May and August 2024; Supplementary Figure S2: Temporal variation in microbial alpha diversity indices across all stations and depths. Supplementary Figure S3: NMDS of microbial beta diversity patterns based on Bray-Curtis similarity; Supplementary Figure S4: Microbial community composition and core microbiome analysis across stations; Supplementary Figure S5: Alpha diversity of ichthyofauna across Sampling, Depth and Station; Supplementary Table S1: Metadata of seawater samples collected from the Thracian Sea. Sample names follow the format SxRyDz, where S denotes the sampling month (S1: May, S2: June, S3: July, S4: August), R refers to the sampling station (R1–R4), and D indicates the depth of sampling (A: surface, B: thermocline). (e.g., S1R1A refers to a surface sample collected in May from station R1.); Supplementary Table S2: Variation of microbial alpha diversity metrics and multivariate analysis (Kruskal-Wallis test) of seawater samples collected from the study area between May and August 2024; Supplementary Table S3: Pairwise comparisons and overall effects for microbial alpha diversity metrics (Shannon Entropy and Pielou Evenness) across sampling months, stations, and depths. Statistical significance was assessed using Dunn’s post hoc test for pairwise comparisons and the Kruskal–Wallis test for overall group differences; Supplementary Table S4: Relative abundance (%) of fish species identified in the samples, presented at species level.; Supplementary Table S5: Summary of topological metrics describing the microbial–eukaryotic co-occurrence networks at the surface and thermocline layers. The surface network exhibited higher complexity and connectivity, as indicated by the increased number of nodes and edges, clustering coefficient, and betweenness centrality; Supplementary Table S6: Metadata associated with the seawater samples collected in the Thracian Sea. The table provides environmental and physicochemical parameters measured for each replicate; Supplementary Table S7: Amplicon Sequence Variant (ASV) table of microbial communities at taxonomic level 6 (genus) derived from 16S rRNA (V4 region) sequencing of seawater samples from the Thracian Sea. Data were normalized. Rows represent samples and columns correspond to bacterial genera. Values indicate normalized relative abundances.; Supplementary Table S8: Amplicon Sequence Variant (ASV) table of ichthyofaunal communities at taxonomic level 7 (species) derived from mtCytB sequencing of seawater samples from the Thracian Sea. Data were normalized. Rows represent samples and columns correspond to taxa. Values indicate normalized relative abundances..
