# Supplementary material for: Spatiotemporal Dynamics of Microbial and Fish Communities in the Thracian Sea Revealed by eDNA Metabarcoding
Source: Microorganisms. 2025 Oct 15;13(10):2373. doi: 10.3390/microorganisms13102373 (PMC12566333; doi:10.3390/microorganisms13102373)
Supplement: Supplementary file 1 [file microorganisms-13-02373-s001.zip › Tokamani-2025-SI.pdf]

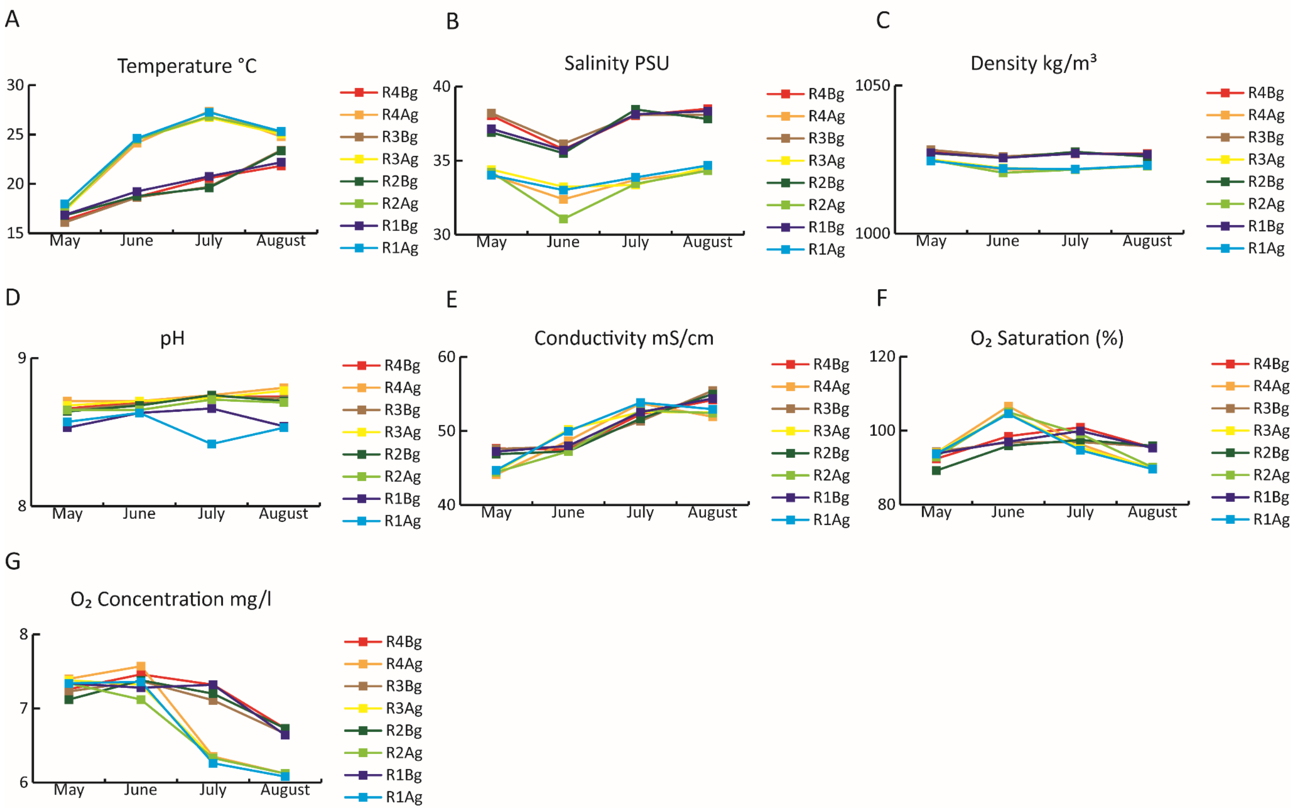

Supplementary Figure S1. Seasonal variation of physicochemical parameters of seawater samples collected from the study area between May and August 2024. *(A) Surface water temperature (°C) increased progressively from May to August, with maximum values observed at the summer peak. (B) Salinity (PSU) exhibited a transient decline in June, followed by a stabilization at higher values during July and August. (C) Seawater density (kg/m³) mirrored changes in temperature and salinity, decreased during June, and increased again towards August. (D) pH values remained relatively stable across months (8.5–8.8), with minor variations between surface and thermocline samples. (E) Conductivity (mS/cm) followed a similar trend, reaching maximum levels by August, due to intensified evaporation and increased salinity. (F) Oxygen saturation (%) peaked in June, suggesting enhanced primary photosynthetic productivity or cooler temperatures, and subsequently declined through August. (G) Oxygen concentration (mg/L) decreased steadily from May to August, reflecting reduced solubility associated with rising temperatures.*

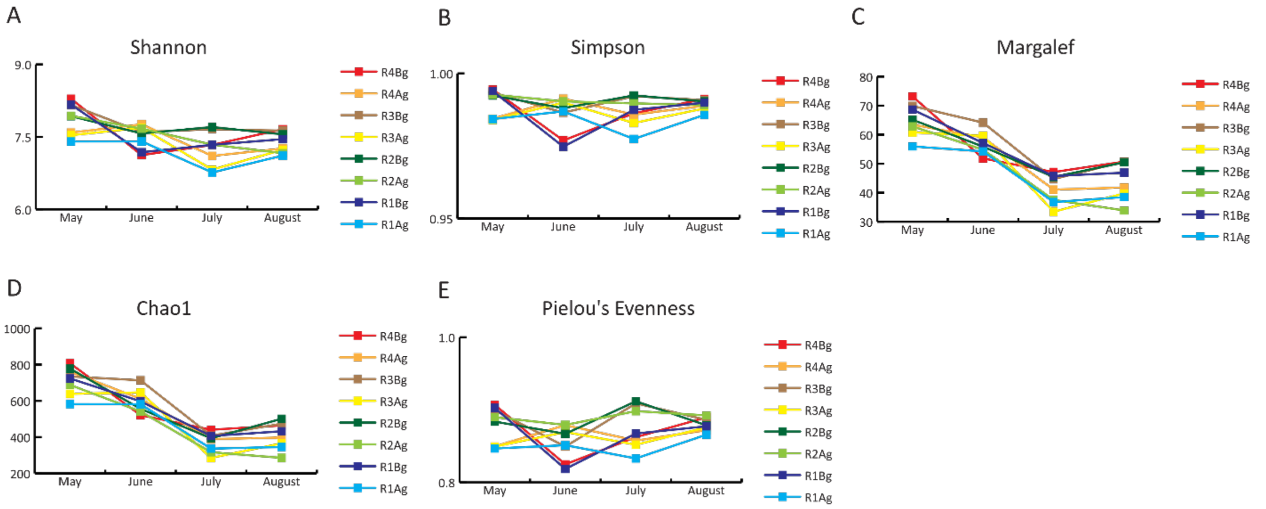

Supplementary Figure S2. Temporal variation in microbial alpha diversity indices across all stations and depths. *Line plots illustrate monthly changes in five diversity metrics for each sampling unit (combination of station and depth): (A) Shannon entropy, (B) Simpson diversity index, (C) Margalef richness, (D) Chao1 estimated richness, and (E) Pielou's evenness. Each line represents a single station–depth combination (e.g., R1Ag, R2Bg [A- Surface, B-Thermocline]), revealing trends in diversity dynamics across four months (May to August). Notably, richness-based metrics such as Shannon, Margalef and Chao1 (A, C, D) showed a steady decline over time, while evenness and entropy-based metrics (B, E) displayed more variable patterns.*

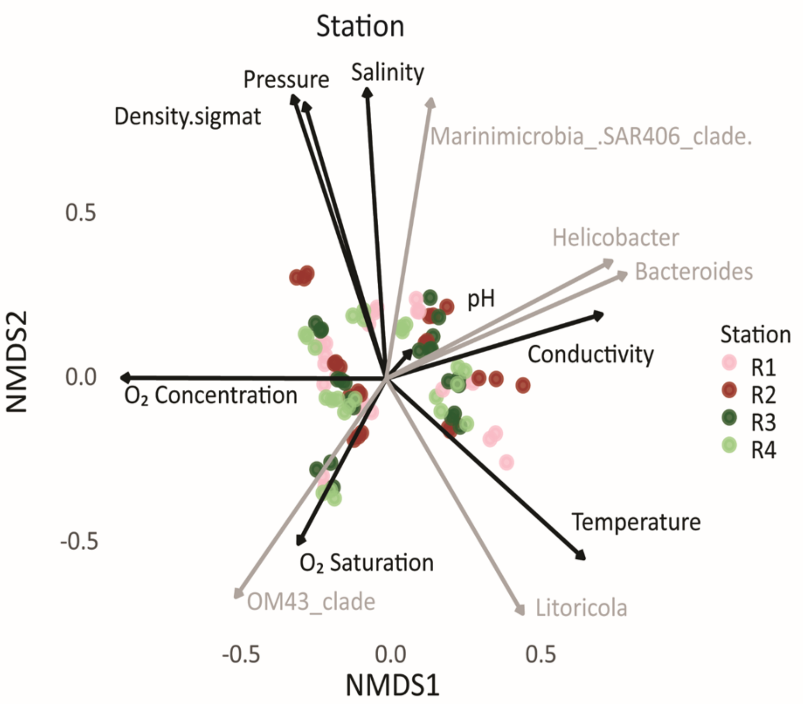

Supplementary Figure S3. NMDS of microbial beta diversity patterns based on Bray-Curtis similarity. *Samples colored by Station (sites R1-R4). Spatial clustering is less pronounced compared to temporal and depth structuring, but localized differences are still evident. The lengths and directions of the fitted vectors represent the strength and direction of correlation with the ordination configuration of environmental parameters. The top five ASVs with the longest vector lengths were also identified and labeled.*

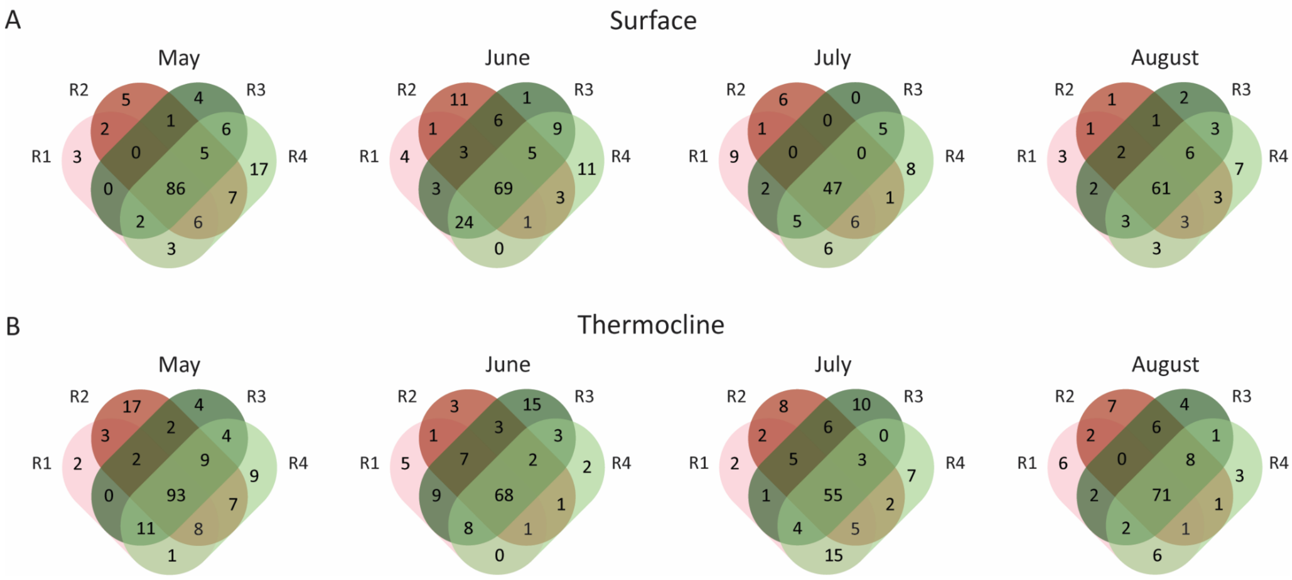

Supplementary Figure S4. Microbial community composition and core microbiome analysis across stations. *Venn diagrams showing the shared and unique species across Stations (R1-R4) for sampling months (May–August) at surface samples (A). Equivalent Venn diagram for thermocline samples, with overlapping species among all stations (B).*

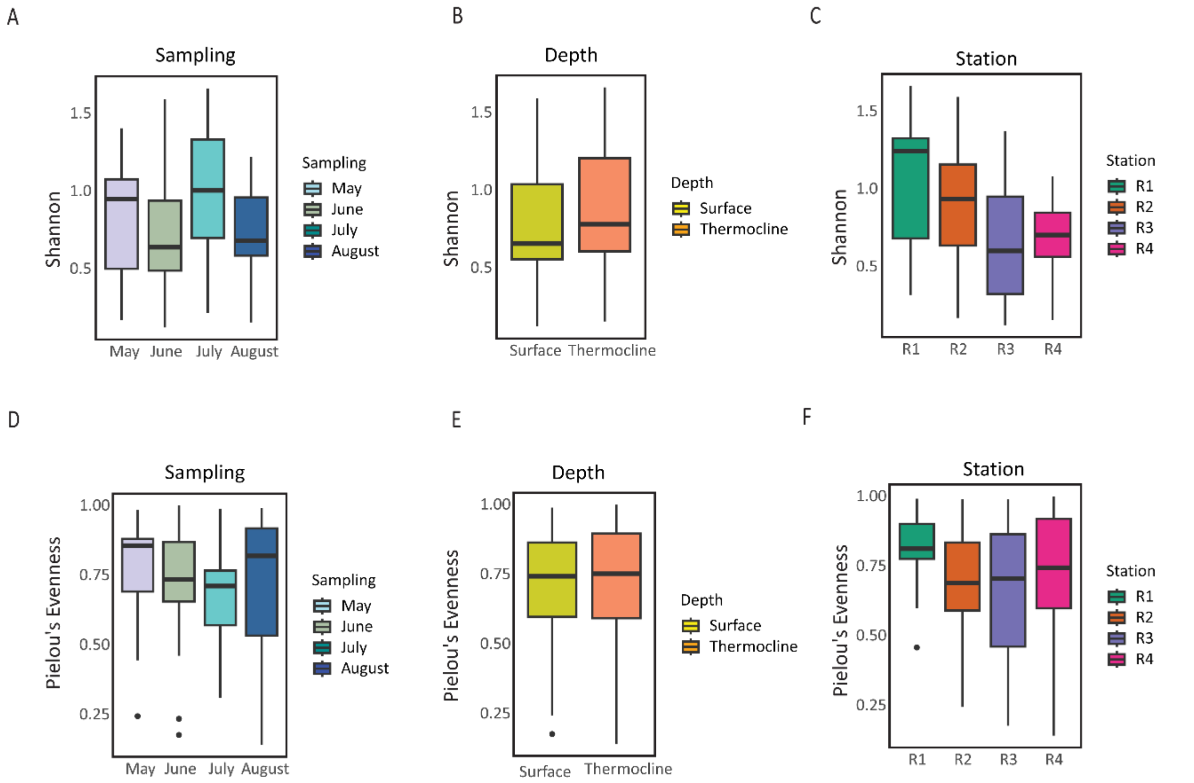

Supplementary Figure S5. Alpha diversity of ichthyofauna across Sampling, Depth, and Station. (A–C) *Boxplots showing variation in Shannon entropy across (A) Sampling (May, June, July, August), (B) Depth (surface vs. thermocline), and (C) Stations (R1–R4). (D–F) Boxplots showing variation in Pielou's evenness across (D) Sampling, (E) Depth, and (F) Stations. Statistical significance was assessed using the Kruskal-Wallis test and Dunn's test. No statistically significant ($p < 0.05$) seasonal and spatial differences were observed.*

Suppl. Table S1. Metadata of seawater samples collected from the Thracian Sea. Sample names follow the format SxRyDz, where S denotes the sampling month (S1: May, S2: June, S3: July, S4: August), R refers to the sampling station (R1–R4), and D indicates the depth of sampling (A: surface, B: thermocline). (e.g., S1R1A refers to a surface sample collected in May from station R1.)

Name	Sampling-Date	Sampling	Station	Depth	Depth (m)	Depth of the station (m)	pH	O_2 concentration (mg/L)	O_2 Saturation (%)	Temperature (^oC)	Salinity (PSU)	Conductivity (mS/cm)	Density (Kg/m^3)
S1R1A	16.05.2024	May	R1	Surface	1	20	8.57	7.34	93.7	17.9686	34.0140	44.711674	1024.5251
S1R1B	16.05.2024	May	R1	Thermocline	17	20	8.53	7.34	93.8	16.8645	37.1485	47.212610	1027.2728
S1R2A	16.05.2024	May	R2	Surface	1	35	8.65	7.34	92.9	17.4334	34.2280	44.437117	1024.8191
S1R2B	16.05.2024	May	R2	Thermocline	20	35	8.64	7.12	89.2	16.7962	36.9080	46.870527	1027.1176
S1R3A	16.05.2024	May	R3	Surface	1	35	8.68	7.38	94.1	17.2508	34.3975	44.453923	1024.9927
S1R3B	16.05.2024	May	R3	Thermocline	20	35	8.65	7.23	94.3	16.0921	38.2069	47.580674	1028.2841
S1R4A	16.05.2024	May	R4	Surface	1	21	8.71	7.40	94.0	17.3519	34.0243	44.121216	1024.6823
S1R4B	16.05.2024	May	R4	Thermocline	17	21	8.66	7.26	92.4	16.3329	38.0433	47.656137	1028.1017
S2R1A	04.06.2024	June	R1	Surface	1	20.5	8.63	7.36	104.5	24.6105	33.0060	49.985153	1021.9531
S2R1B	04.06.2024	June	R1	Thermocline	12	20.5	8.63	7.28	97.0	19.2315	35.7111	47.996822	1025.5583
S2R2A	04.06.2024	June	R2	Surface	1	34	8.65	7.12	105.0	24.4951	31.0636	47.242420	1020.5201
S2R2B	04.06.2024	June	R2	Thermocline	12	34	8.68	7.38	95.9	18.7541	35.4975	47.252981	1025.5168
S2R3A	04.06.2024	June	R3	Surface	1	34	8.71	7.32	104.6	24.5242	33.2321	50.204267	1022.1495
S2R3B	04.06.2024	June	R3	Thermocline	12	34	8.69	7.37	96.8	18.6277	36.1405	47.883789	1026.0420
S2R4A	04.06.2024	June	R4	Surface	1	20.5	8.71	7.57	106.6	24.1302	32.3895	48.684111	1021.6290
S2R4B	04.06.2024	June	R4	Thermocline	12	20.5	8.70	7.46	98.5	18.6558	35.7578	47.460656	1025.7413
S3R1A	25.07.2024	July	R1	Surface	1	20	8.42	6.26	94.7	27.2514	33.8669	53.838320	1021.7850
S3R1B	25.07.2024	July	R1	Thermocline	17	20	8.66	7.32	99.9	20.7539	38.1267	52.540899	1027.0190
S3R2A	25.07.2024	July	R2	Surface	1	35	8.72	6.33	99.1	26.8217	33.4332	52.789054	1021.5959
S3R2B	25.07.2024	July	R2	Thermocline	20	35	8.75	7.20	97.5	19.6028	38.4537	51.671430	1027.5921
S3R3A	25.07.2024	July	R3	Surface	1	30	8.73	6.33	95.4	26.6999	33.3595	52.562216	1021.5781
S3R3B	25.07.2024	July	R3	Thermocline	20	30	8.74	7.11	96.9	19.6977	38.0884	51.339264	1027.2880
S3R4A	25.07.2024	July	R4	Surface	1	20	8.75	6.35	96.3	27.3602	33.7109	53.729022	1021.6329
S3R4B	25.07.2024	July	R4	Thermocline	17	20	8.74	7.32	100.9	20.5805	38.0420	52.247182	1027.0014
S4R1A	12.09.2024	August	R1	Surface	1	20	8.53	6.08	89.6	25.3074	34.6744	52.950703	1023.0023
S4R1B	12.09.2024	August	R1	Thermocline	14	20	8.54	6.64	95.3	22.1814	38.3455	54.389581	1026.7713
S4R2A	12.09.2024	August	R2	Surface	1	32	8.70	6.12	90.1	25.2532	34.3234	52.418031	1022.7537
S4R2B	12.09.2024	August	R2	Thermocline	14	32	8.71	6.73	95.9	23.3399	37.8116	54.996480	1026.0285
S4R3A	12.09.2024	August	R3	Surface	1	32	8.78	6.12	90.0	25.0357	34.5533	52.505317	1022.9935
S4R3B	12.09.2024	August	R3	Thermocline	14	32	8.73	6.66	95.7	23.4154	38.0950	55.445631	1026.2218
S4R4A	12.09.2024	August	R4	Surface	1	20	8.80	6.12	89.6	24.7743	34.3230	51.925647	1022.8981
S4R4B	12.09.2024	August	R4	Thermocline	14	20	8.74	6.73	95.4	21.8169	38.5072	54.185831	1026.9984

Suppl. Table S2. Variation of microbial alpha diversity metrics and multivariate analysis (Kruskal-Wallis test) of seawater samples collected from the study area between May and August 2024.

Metric	n	statistic	df	P	Group
Shannon entropy	96	32.404	3	0.00000043	Month
	96	12.012	1	0.000528	Depth
	96	8.257	3	0.041	Station
Pielou' evenness	96	15.048	3	0.00178	Month
	96	7.958	1	0.00479	Depth
	96	17.359	3	0.000596	Station
Margalef	96	69.115	3	6.6E-15	Month
	96	8.421	1	0.00371	Depth
	96	1.062	3	0.786	Station
Simpson	96	11.896	3	0.00775	Month
	96	10.921	1	0.000951	Depth
	96	13.636	3	0.00344	Station
Chao1	96	67.524	3	1.45E-14	Month
	96	4.994	1	0.0254	Depth
	96	1.539	3	0.673	Station

Suppl Table S3. Pairwise comparisons and overall effects for microbial alpha diversity metrics (Shannon Entropy and Pielou Evenness) across sampling months, stations, and depths. Statistical significance was assessed using Dunn's post hoc test for pairwise comparisons and the Kruskal–Wallis test for overall group differences.

Group	Comparison	Z	P.unadj	P.adj	Metric	Method
Sampling	August - July	0.9896	0.3223	0.3223	Shannon Entropy	Dunn test
Sampling	August - June	-1.4507	0.1468	0.1762	Shannon Entropy	Dunn test
Sampling	July - June	-2.4404	0.0146	0.022	Shannon Entropy	Dunn test
Sampling	August - May	-4.3472	1.3788	4.1364E-05	Shannon Entropy	Dunn test
Sampling	July - May	-5.3368	9.457	5.6742E-07	Shannon Entropy	Dunn test
Sampling	June - May	-2.8964	0.0037	0.0075	Shannon Entropy	Dunn test
Station	R1 - R2	-2.7046	0.0068	0.041	Shannon Entropy	Dunn test
Station	R1 - R3	-2.1917	0.0283	0.0851	Shannon Entropy	Dunn test
Station	R2 - R3	0.5129	0.6079	0.6079	Shannon Entropy	Dunn test
Station	R1 - R4	-1.5906	0.1116	0.2233	Shannon Entropy	Dunn test
Station	R2 - R4	1.114	0.2652	0.3979	Shannon Entropy	Dunn test
Station	R3 - R4	0.6010	0.5478	0.6573	Shannon Entropy	Dunn test
Sampling	August - July	1.2642	0.2061	0.3092	Pielou Evenness	Dunn test
Sampling	August - June	3.4974	0.0004	0.0028	Pielou Evenness	Dunn test
Sampling	July - June	2.2331	0.0255	0.0510	Pielou Evenness	Dunn test
Sampling	August - May	0.2953	0.7677	0.7677	Pielou Evenness	Dunn test
Sampling	July - May	-0.9689	0.3325	0.3990	Pielou Evenness	Dunn test
Sampling	June - May	-3.2021	0.0013	0.0040	Pielou Evenness	Dunn test
Station	R1 - R2	-4.0881	4.3484E-05	0.0002	Pielou Evenness	Dunn test
Station	R1 - R3	-2.0622	0.0391	0.0783	Pielou Evenness	Dunn test
Station	R2 - R3	2.0259	0.0427	0.0641	Pielou Evenness	Dunn test
Station	R1 - R4	-1.3938	0.1633	0.1960	Pielou Evenness	Dunn test
Station	R2 - R4	2.6943	0.0070	0.0211	Pielou Evenness	Dunn test
Station	R3 - R4	0.6684	0.5038	0.5038	Pielou Evenness	Dunn test
**Group**	**Comparison**	**statistic**	**df**	**p**	**Metric**	**Method**
Depth	Surface -Thermocline	12.0129	1	0.0005	Shannon Entropy	Kruskal-Wallis
Depth	Surface -Thermocline	7.9588	1	0.0047	Pielou Evenness	Kruskal-Wallis

Suppl. Table S5 . Summary of topological metrics describing the microbial–eukaryotic co-occurrence networks at the surface and thermocline layers. The surface network exhibited higher complexity and connectivity, as indicated by the increased number of nodes and edges, clustering coefficient, and betweenness centrality.

Metric	Surface Network	Thermocline Network
**Number of Nodes**	179	135
**Number of Edges**	629	309
**Density**	0.0394	0.0341
**Average Degree**	7.03	4.58
**Average Shortest Path**	3.41	3.52
**Network Diameter**	9	8
**Clustering Coefficient**	0.41	0.35
**Mean Closeness**	0.00283	0.00296
**Mean Betweenness**	72.1	40.6
**Connected Network**	Yes	Yes
